# Prognostic Influence of Spontaneous Tumor Rupture in Patients With Hepatocellular Carcinoma After Hepatectomy: A Meta-Analysis of Observational Studies

**DOI:** 10.3389/fsurg.2021.769233

**Published:** 2021-11-16

**Authors:** Jiaxuan Xu, Jiaze Hong, Yiran Wang, Lingling Zhou, Binbin Xu, Yuexiu Si, Yujing He, Yizhou Chen

**Affiliations:** ^1^The Second Clinical Medical College, Zhejiang Chinese Medical University, Hangzhou, China; ^2^The First Clinical Medical College, Wenzhou Medical University, Wenzhou, China; ^3^Department of Nutrition, HwaMei Hospital, University of Chinese Academy of Sciences, Ningbo, China; ^4^School of Basic Medical Sciences, Zhejiang Chinese Medical University, Hangzhou, China; ^5^Emergency Medical Center, Ningbo Yinzhou No. 2 Hospital, Ningbo, China

**Keywords:** hepatocellular carcinoma, spontaneous tumor rupture, hepatectomy, prognosis, meta-analysis

## Abstract

**Objective:** This study aims to comprehensively analyze the influence of spontaneous tumor rupture on the prognosis of hepatocellular carcinoma patients following hepatic resection.

**Methods:** We systematically searched four online electronic databases, including PubMed, Embase, Web of Science, and Cochrane Library, for eligible studies published from inception to March 2021. The main endpoints were overall survival (OS) and disease-free survival (DFS).

**Results:** This meta-analysis included 21 observational articles with 57,241 cases. The results revealed that spontaneous tumor rupture was associated with worse OS (hazard ratio (HR), 1.65; 95% confidence interval (CI), 1.33–2.05) and DFS (HR, 1.42; 95% CI, 1.12–1.80) in resectable hepatocellular carcinoma patients. This phenomenon was observed in most subgroups, which were classified by recorded survival time, age, country, alpha-fetoprotein (AFP) concentration, liver cirrhosis, and microvascular invasion. However, in subgroups of macrovascular invasion positive, spontaneous tumor rupture was not a risk factor for OS (HR, 1.55; 95% CI, 0.99–2.42) and DFS (HR, 1.23; 95% CI, 0.91–1.65) in hepatocellular carcinoma patients after hepatectomy. For macrovascular invasion negative, compared with non-ruptured hepatocellular carcinoma patients, ruptured hepatocellular carcinoma patients exhibited worse prognosis for OS (HR, 1.55; 95% CI, 0.99–2.42) and DFS (HR, 1.23; 95% CI, 0.91–1.65) following hepatectomy.

**Conclusions:** Spontaneous tumor rupture was a prognostic risk factor for hepatocellular carcinoma patients after hepatic resection. However, in macrovascular invasion patients, spontaneous tumor rupture was not a prognostic risk factor.

## Introduction

Hepatocellular carcinoma (HCC), the sixth most prevalent primary neoplasm, was responsible for around 81,0000 deaths in 2015 worldwide (1, 2). Spontaneous tumor rupture (STR) of HCC is a potentially fatal complication (3). The mechanisms underlying STR remain unclear. Possible reasons include large tumor size, ischemic necrosis, and vascular compression caused by rapid tumor growth (4–6). Although the overall incidence was relatively low (3–26%), the mortality rates of ruptured HCC patients were extremely high (32–75%) in reported literature (3, 7–11). Nowadays, treating STR of HCC is challenging; the current interventions used clinically include conservative treatment, transcatheter arterial chemoembolization (TACE), and hepatic resection (12, 13). Hepatectomy, including emergent and staged (after TACE achieving hemostasis) hepatectomy, provided a better long-term prognosis than palliative treatment in ruptured HCC patients with relatively well-preserved liver functions (13).

Traditionally, STR has recognized as a terminal event of HCC, as it could lead to various symptoms, such as hemorrhagic shock, intraperitoneal hemorrhage, and metastases, and most ruptured HCC patients had portal vein tumor thrombosis (PVTT), impaired liver function, and liver cirrhosis (14–17). As a result, these advanced patients with STR were frequently unable to receive surgical treatment and were compelled to have non-surgical treatment, resulting in a worse long-term prognosis than advanced patients receiving the same therapy without STR (9, 12).

However, whether STR was a prognostic risk factor for HCC patients after hepatic resection remains unclear (18, 19). Consequently, this meta-analysis aims to evaluate the long-term prognosis of patients with or without STR following hepatectomy and explore whether STR affects the prognosis of HCC patients after surgery.

## Materials and Methods

### Literature Search Strategy

This meta-analysis followed Preferred Reporting Items for Systematic Review and Meta-Analysis guidelines (20). Four online electronic databases (PubMed, Embase, Web of Science, and Cochrane Library) were searched for published literature in English from inception to March 2021. The search strategies included: (“Hepatocellular Carcinoma” OR “Hepatoma” OR “Liver Cell Carcinomas” OR “HCC”) AND (“Rupture”). Furthermore, potentially eligible studies were identified through a thorough inspection from reference lists of all retrieved papers.

### Inclusion Criteria

The inclusion criteria for this meta-analysis entailed: (1) Patients in experiment (ruptured HCC) and control (non-ruptured HCC) groups received hepatic resection, including emergent and staged hepatectomy. (2) The included literature is original and includes observational studies (OBSs). (3) The study evaluated the relationship between tumor rupture and prognosis. (4) The primary endpoints as overall survival (OS) or disease-free survival (DFS) were mentioned, and their hazard ratio (HR) and 95% confidence interval (CI) were obtainable or could be calculated.

### Exclusion Criteria

The exclusion criteria for this meta-analysis entailed: (1) The relationship between ruptured and non-ruptured HCC in the prognosis of patients has not been explored at the same time. (2) When the duplicate publications were reviewed, the higher-quality or most updated was included. (3) The intervention for patients was not surgery but like TACE alone and palliative chemotherapy. (4) Multiple hepatic metastases, distant organ metastasis, and lymph node metastases were found in patients. (5) The tumor rupture was not spontaneous, but it was caused by trauma.

### Data Extraction and Quality Evaluation

Based on pre-determined inclusion/exclusion criteria, two authors performed an independent review, extracting the following information carefully from each included study, including (1) study characteristics (author, country, and publication year), (2) patients' basic characteristics (age, gender, and number of included patients), (3) hepatic features (serum AFP, virus status, and liver cirrhosis), (4) tumor features (tumor number, size, and invasion), (5) therapeutic effect (OS and DFS, and corresponding HR and 95% CI).

The quality of incorporated OBSs was assessed using Newcastle-Ottawa Scale (NOS) that encompassed three aspects (selection of patients, comparability of groups, and evaluation of outcomes). The cumulative scores of articles less than six were considered of low-quality (21).

### Statistical Analysis

The pooled HR and 95% CI for OS and DFS were calculated to estimate the relationship between tumor rupture and prognosis. Heterogeneity among included literature was assessed using I^2^ statistic. For potential heterogeneity, random-effect models were employed for greater reliability. When the number of included articles in each analysis is ≥ 10, Egger's test based on Stata 12.0 software was conducted to evaluate publication bias (22). A sensitivity analysis was conducted to determine robustness of conclusions. *P*-value <0.05 was considered statistically significant.

## Results

### Data Collection and Characteristics

**Figure 1:**
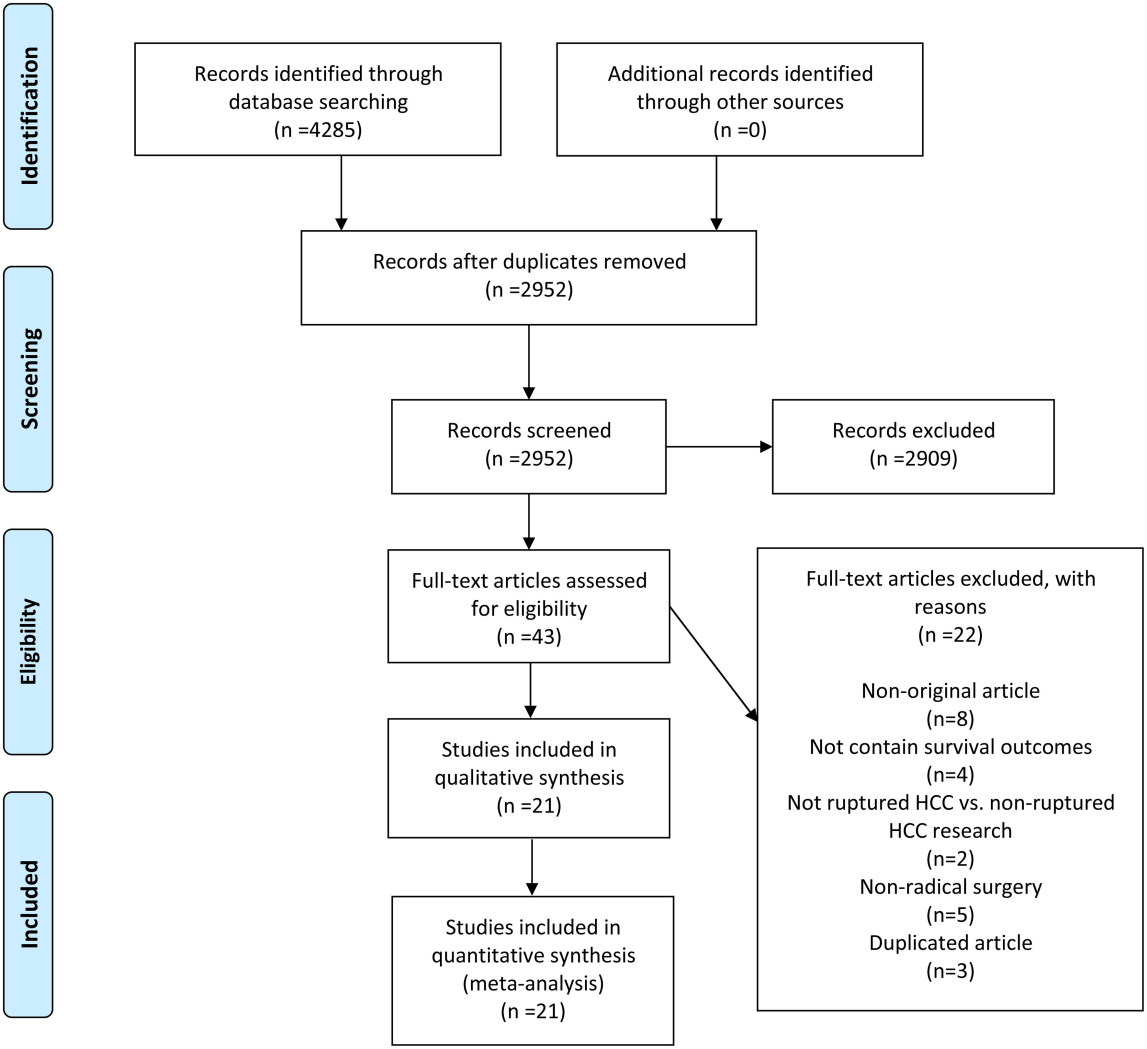
A schematic flow for selecting the articles included in this meta-analysis.

A total of 4,285 records were initially yielded from four electronic databases using a pre-designed search strategy. After removing duplicates, 2,952 records remained. Twenty-one studies (13, 18, 19, 23–40) were ultimately included following a strict screening process. The comprehensive literature review and rigorous selection process are displayed in Figure 1.

A total of 57,241 patients were enrolled in 21 OBSs mainly originated from Asia (19/21), followed by South America (1/21) and Europe (1/21). Eight studies simultaneously analyzed OS and DFS, ten were with OS alone, and three were only related to DFS. The detailed patients' characteristics of demographic and clinicopathological aspects are shown in Table 1 and Supplementary Table 1. The quality of OBSs was assessed using NOS and assessment outcomes indicated that incorporated articles were of high quality (Supplementary Table 2).

**Table 1:** Characteristics of all the studies included in the meta-analysis.

**Author**	**Country**	**Number of patients**		**AFP(ng/ml)**		**Liver cirrhosis (number)**		**Microvascular invasion (number)**		**Macrovascular invasion (number)**	
		**Rupture**	**Non-rupture**	**Rupture**	**Non-rupture**	**Rupture**	**Non-rupture**	**Rupture**	**Non-rupture**	**Rupture**	**Non-rupture**
Aoki et al. (13)	Japan	1160	48548	510 cases≥400 504 cases <400	9,279 cases≥400 36,431 cases <400	579	26,473	NA	NA	352	5,373
Chan et al. (19)	China	84	1,254	472	91	43	750	52	583	9	102
Cheng et al. (23)	China	53	826	66.8		507		104		188	
Chua et al. (18)	Singapore	49	98	NA	NA	19	32	27	47	7	11
Fan et al. (24)	China	211		NA	NA	98		NA	NA	21	
Joliat et al. (25)	Switzerland	14	126	23	18	11	81	NA	NA	NA	NA
Kwon et al. (26)	Korea	85	186	1,2151	14,908	NA	NA	29	58	7	13
Lee et al. (27)	Korea	18	37	NA	NA	11	21	12	26	8	13
Li et al. (28)	China	89	171	138.5	73.8	73	99	0	0	NA	NA
Miyoshi et al. (29)	Japan	10	295	3 cases≥1,000 7 cases <1,000	53 cases≥1,000 242 cases <1,000	NA	NA	NA	NA	6	81
Mizuno et al. (30)	Japan	6	15	3 cases>400 3 cases <400	5 cases>400 10 cases <400	NA	NA	NA	NA	5	6
Ruan et al. (31)	China	57	57	22	20	28	NA	23	21	NA	NA
Ruiz et al. (32)	Peru	253		2,651		39		64		29	
Tanaka et al. (33)	Japan	42	42	78.8	49.5	13	14	NA	NA	2	3
Uchiyama et al. (34)	Japan	27	1,004	168 cases≥400 836 cases <400		NA	NA	NA	NA	40	
Xiao et al. (35)	China	53	181	141 cases≥400 93 cases <400		196		144		234	
Yang et al. (36)	China	143	1,090	85 cases≥400 58 cases <400	420 cases≥400 670 cases <400	NA	NA	97	668	36	172
Yeh et al. (37)	China	35	175	100 cases≥400 110 cases <400		63		NA	NA	116	
Zhang et al. (38)	China	41	446	5788.9	3947.1	24	285	NA	NA	1	48
Zhao et al. (39)	China	12	70	35 cases≥400 47 cases <400		65		19		0	0
Zhu et al. (40)	China	89	89	57 cases≥400 32 cases <400	53 cases≥400 36 cases <400	76	80	65	60	50	43

*AFP, a-fetoprotein; NA, not available*.

### Effect of STR on OS and DFS

**Figure 2:**
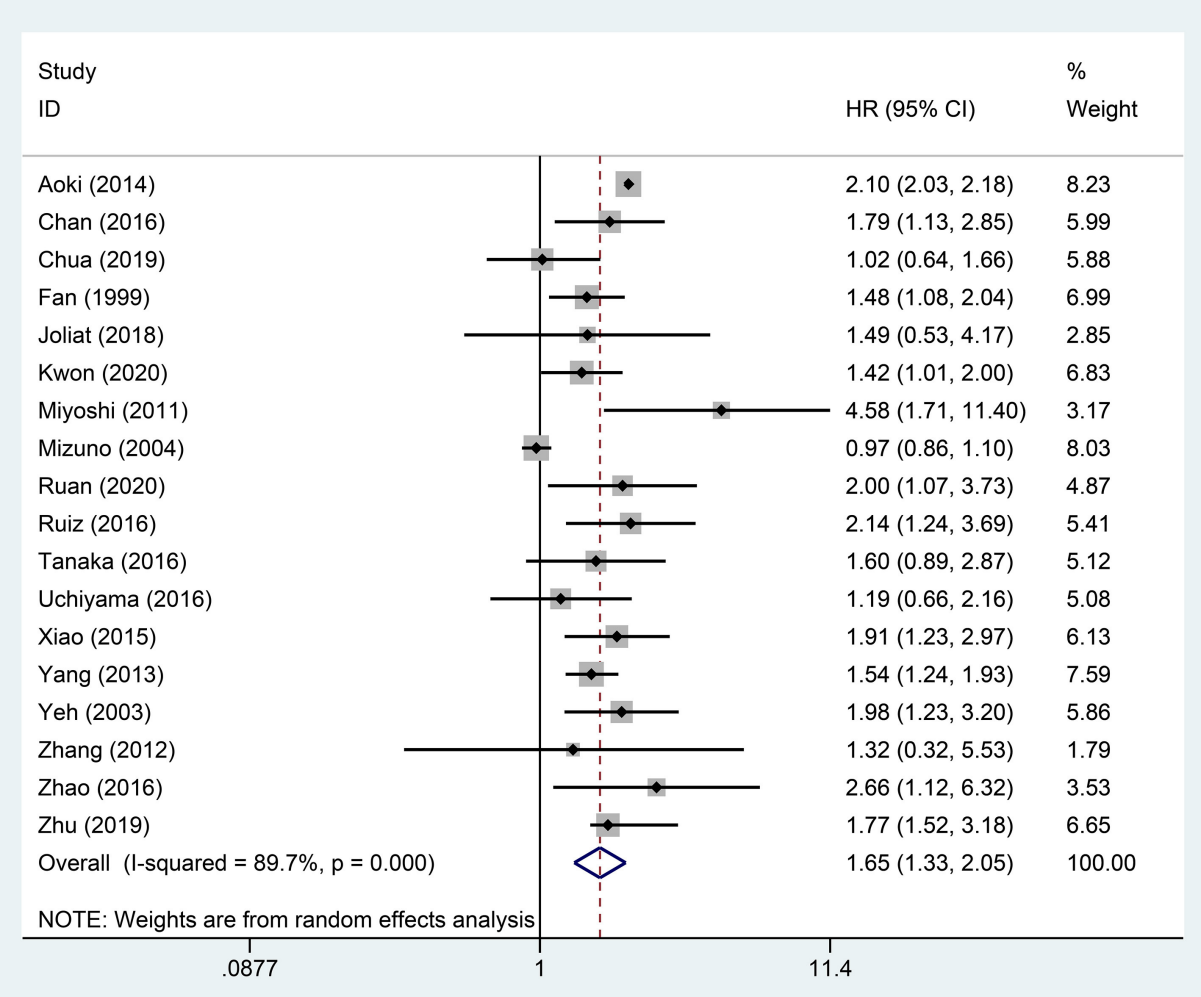
Forest plot of OS of ruptured hepatocellular carcinoma (HCC) patients after hepatectomy (*P* <0.001).

**Figure 3:**
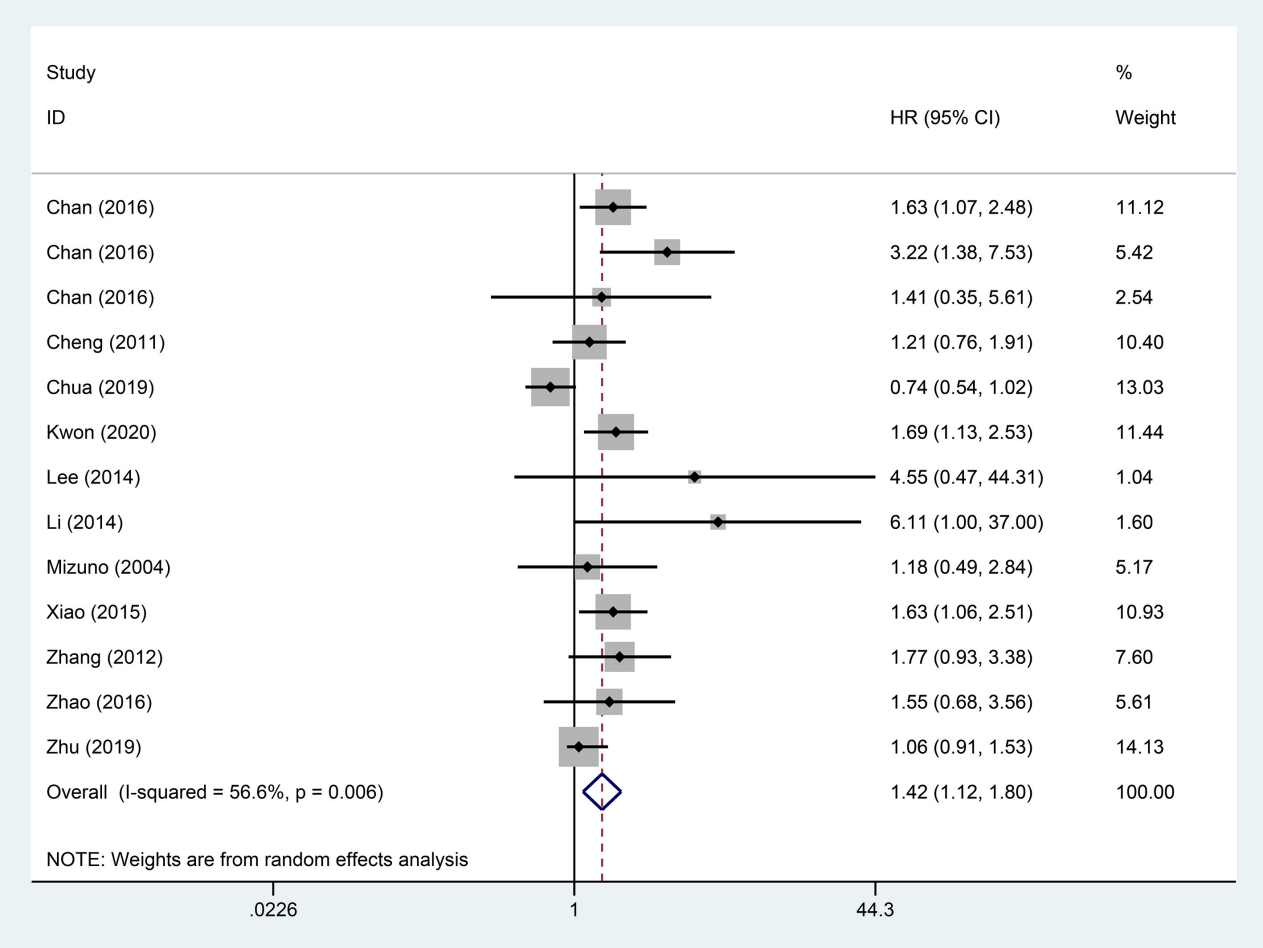
Forest plot of DFS of ruptured hepatocellular carcinoma (HCC) patients following hepatectomy (*P* = 0.004).

A pooled analysis based on 18 studies including relevant OS data exhibited that STR was potentially related to a worse prognosis of ruptured HCC patients (HR, 1.65; 95% CI, 1.33–2.05) (Figure 2). Consistent with the pooled result of OS, pooled DFS outcomes also illustrated that ruptured HCC patients had a poorer prognosis than non-ruptured HCC patients (HR, 1.42; 95% CI, 1.12–1.80) (Figure 3).

### Subgroup Analysis

**Figure 4:**
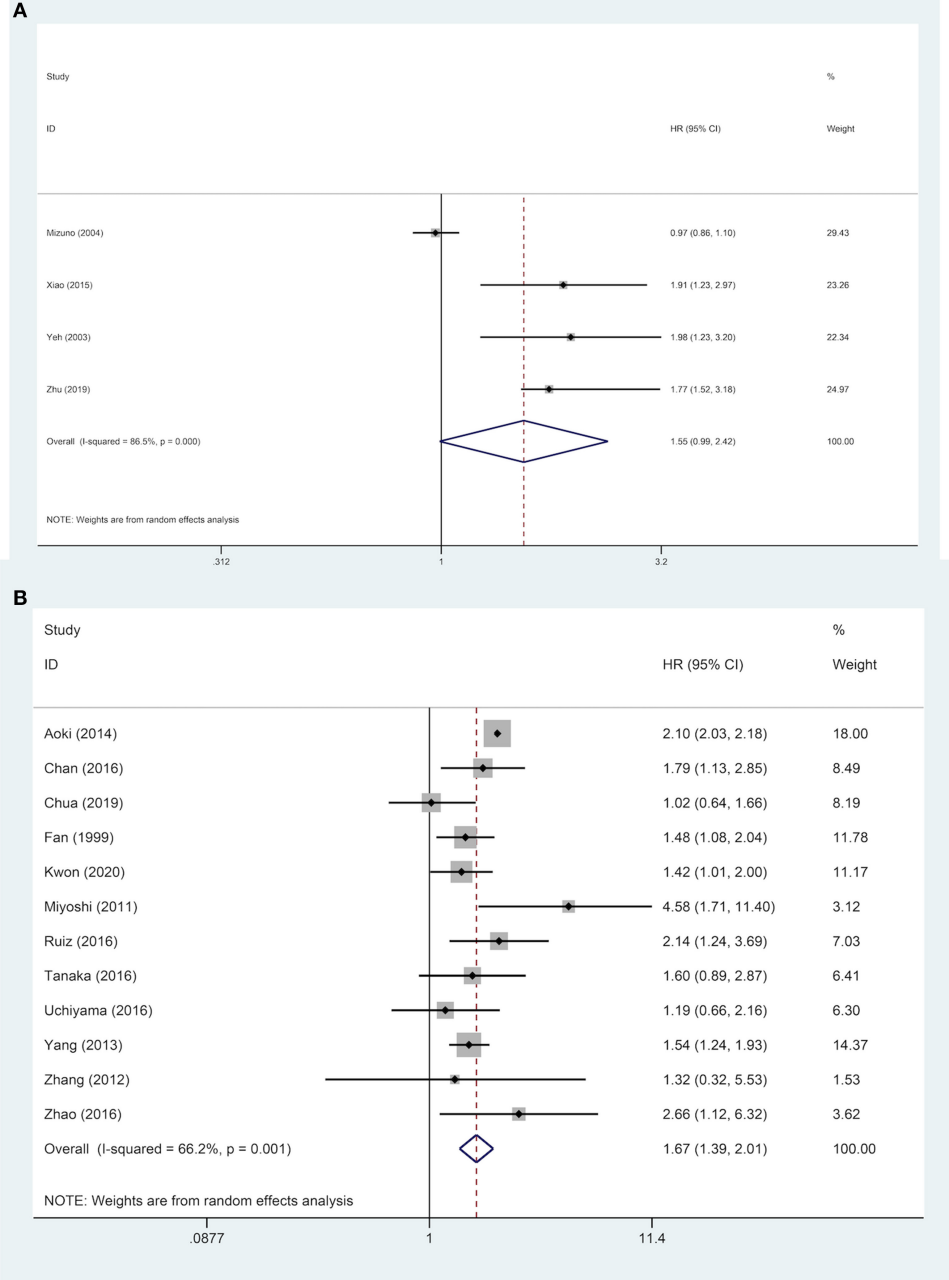
Forest plot of OS of ruptured hepatocellular carcinoma (HCC) patients with macrovascular invasion after hepatectomy (**A**, macrovascular invasion positive, *P* = 0.055; **B**, macrovascular invasion negative, *P* <0.001).

Subgroups analyses were implemented to explore the effect of various factors on the prognosis of ruptured and non-ruptured HCC patients. We categorized the studies into 3-year OS and 5-year OS groups based on recorded survival time. For subgroups of 5-year OS, non-ruptured HCC patients obtained greater OS than ruptured HCC patients, whereas no statistical difference was found in subgroups of 3-year OS. For subgroups of patients' age ≥ or <60 years old, patients in China or other Asian countries, patients' AFP ≥ or <400 ng/mL, patients with and without liver cirrhosis, and patients' microvascular invasion positive/negative, the analysis results all indicated that STR was associated with worse OS (Table 2). For patients with macrovascular invasion positive patients, STR had no adverse impact on ruptured HCC patients' OS compared to non-ruptured HCC patients (HR, 1.55; 95% CI, 0.99–2.42). However, in macrovascular invasion-negative patients, STR was a prognostic risk factor for HCC patients (HR, 1.67; 95% CI, 1.39–2.01) (Figure 4).

**Table 2:** Subgroup analysis of the hepatocellular carcinoma (HCC) rupture on the prognosis of patients with HCC.

	**No. of studies**	**HR**	**95%CI**	**Heterogeneity (*I*^**2**^) (%)**
**Overall survival (OS)**				
3-year OS	3	1.87	0.80–4.39	86.4
5-year OS	15	1.68	1.46–1.94	54.8
Age ≥60 years	5	1.78	1.19–2.65	68.3
Age <60 years	11	1.66	1.47–1.88	0
China	9	1.68	1.47–1.92	0
Non-Chinese Asian countries	7	1.50	1.00–2.26	96.2
AFP≥400 ng/ml	6	1.74	1.45–2.09	0
AFP <400 ng/ml	8	1.58	1.09–2.28	95.3
Liver cirrhosis	7	2.09	2.02–2.17	0
Non-liver cirrhosis	5	1.56	1.23–1.98	26.2
Microvascular invasion positive	4	1.55	1.25–1.91	30.4
Microvascular invasion negative	5	1.74	1.40–2.17	0
**Disease-free survival (DFS)**				
Age <60	9	1.53	1.24–1.88	31.4
China	7	1.50	1.18–1.90	36.7
Non-Chinese Asian countries	4	1.22	0.67–2.24	74.3
AFP≥400 ng/ml	4	1.42	1.07–1.90	49.9
AFP <400 ng/ml	4	1.35	0.93–1.97	3.4
Liver cirrhosis	7	1.52	1.15–2.01	38.5
Microvascular invasion positive	4	1.11	0.74–1.65	70.6
Microvascular invasion negative	5	1.64	1.30–2.06	3.6

*AFP, alpha fetoprotein; HR, hazard ratio; CI, confidence interval*.

**Figure 5:**
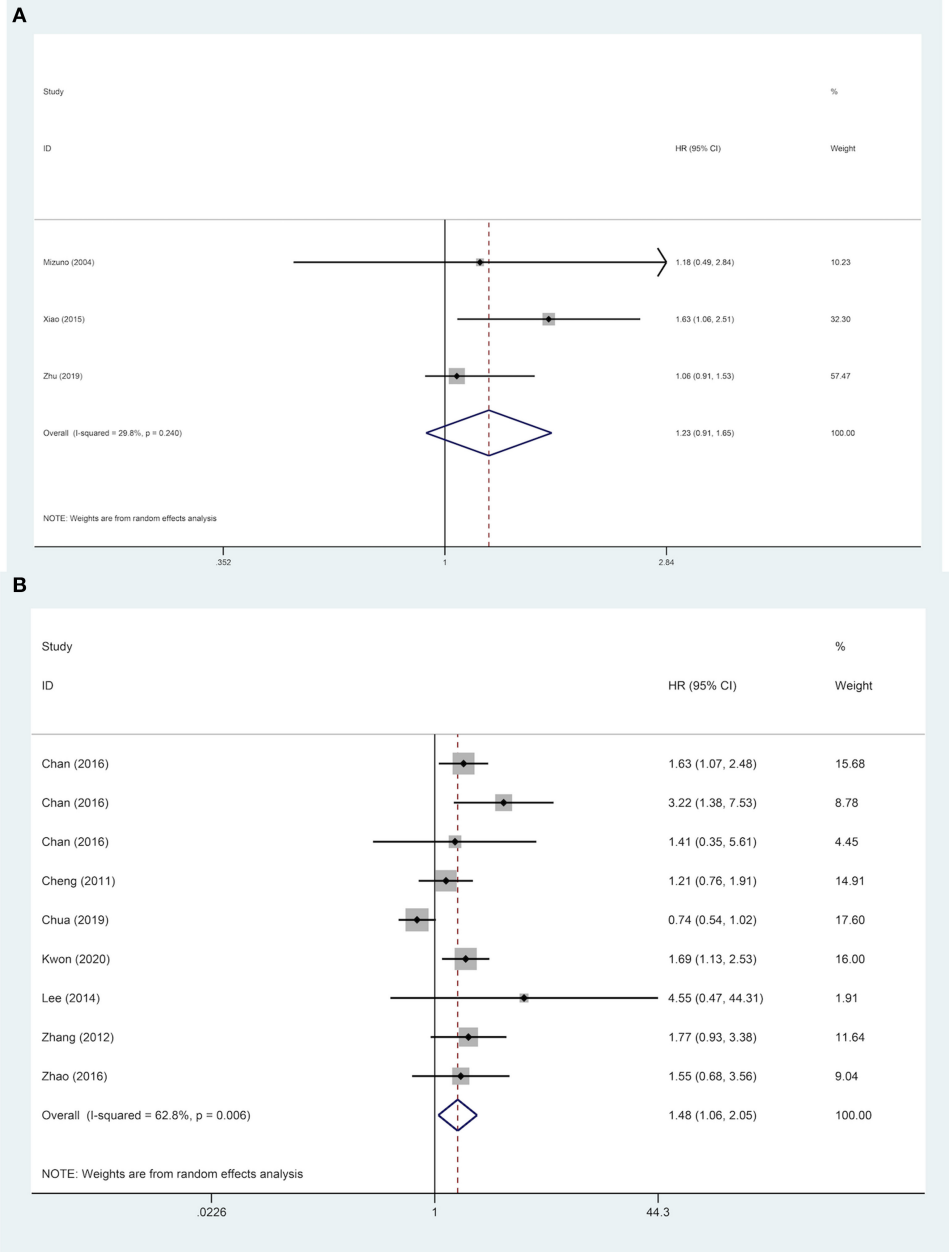
Forest plot of DFS of ruptured hepatocellular carcinoma (HCC) patients with macrovascular invasion after hepatectomy (**A**, macrovascular invasion positive, *P* = 0.170; **B**, macrovascular invasion negative, *P* = 0.021).

Nine studies were included to explore the effect of age on DFS of HCC patients. The results demonstrated that in a subgroup of age <60 years old, ruptured HCC patients' DFS was shorter than in the control group. Although no statistical difference was observed between the two groups' DFS regarding other Asian countries, non-ruptured HCC patients achieved better DFS than ruptured HCC patients in China. When patients' AFP concentration ≥ 400 ng/mL, STR is a potential risk factor for patients' DFS. However, in patients with AFP concentration <400 ng/mL, STR was not correlated with HCC patients' DFS. For patients with liver cirrhosis, STR was linked to worse DFS. Similar poor outcomes were also demonstrated in microvascular invasion-negative patients, but in microvascular invasion-positive patients, no significant difference in DFS was identified between the two groups (Table 2). For DFS of patients, STR was not a prognostic risk factor in macrovascular invasion positive patients (HR, 1.23; 95% CI, 0.91–1.65), but it was a risk factor in macrovascular invasion negative patients (HR, 1.48; 95% CI, 1.06–2.05) (Figure 5).

### Sensitivity Analysis and Publication Bias

After omitting the included articles in sequence, sensitivity analysis results confirmed the excellent stability of HR for OS. The quantificational Egger's test was employed to evaluate publication bias, and the outcomes revealed no potential publication bias among the included articles on HR for OS (*P* > 0.05). Additionally, another sensitivity analysis was performed to verify HR robustness for DFS, resulting in reliable results. No potential publication bias was observed for HR for DFS after Egger's test (*P* > 0.05).

## Discussion

Most ruptured HCC patients were in advanced disease stage; among them, many patients exhibited extrahepatic metastasis, PVTT, and impaired liver function (14–17). These tended to cause them to lose the opportunity of surgery and choose conservative treatment options. Therefore, the traditional concept that STR was a prognostic risk factor for HCC patients was mostly based on receiving non-surgical treatment (9, 12, 41, 42). It is worth investigating whether STR remained a prognostic risk factor for those HCC patients undergoing liver resection. The overall findings from this meta-analysis implied that STR was a risk factor in long-term prognosis of HCC patients following hepatic resection, consistent with previous reports (19, 24, 32).

To thoroughly investigate the reasons of STR affecting long-term prognosis of HCC patients after hepatic resection, from previous literature, we inferred that potential reasons were correlated with gender, tumor size, virus status, hepatectomy style, and liver cirrhosis (13, 18, 24, 28, 37, 43–47). STR was more frequently observed in male patients from reported studies (18, 24). The literature revealed that HCC female patients exhibit a better survival rate and low recurrence rate than male patients (43). Then, it was reported that ruptured HCC patients tended to have larger tumor size than non-ruptured HCC patients, and the total tumor volume is a vital prognostic predictor, and larger HCC was associated with a worse OS and DFS (37, 44). From a nationwide survey (1160 ruptured HCC patients), Aoki et al. (13) found that hepatitis B virus (HBV)-infected patients have a higher STR incidence than hepatitis C virus (HCV)-infected patients. According to reports, long-term survival rates of HCC patients with hepatitis B surface antigen (HBsAg) positive was worse than that of HBsAg negative patients following surgery (45). Additionally, staged hepatectomy followed TACE was a prevalent surgical way for ruptured HCC patients. Hanazaki et al. (46) found that preoperative TACE would significantly increase the risk of patients' postoperative recurrence, leading to unsatisfactory long-term prognosis. Besides, numerous studies revealed that ruptured HCC patients were often accompanied by liver cirrhosis, an independent prognostic risk factor affecting prognosis of HCC patients (28, 47). For reduced liver reserve and tolerance, STR was undoubtedly a serious blow to the disease.

Due to high heterogeneity, the situations of ruptured HCC patients were complicated and diverse. We performed subgroup analyses of the prognosis of HCC patients. The analysis result of 5-year OS subgroup revealed that STR was a risk factor, but no statistical difference in survival was observed between the two groups in 3-year OS subgroup, possibly due to limited sample size (three included studies). In addition, long-term follow-up is required after hepatic resection to determine the difference in prognosis.

Our results indicated that STR was correlated with a poorer prognosis for both patients older and younger than 60 years old. There is still controversy regarding whether age affects tumor recurrence and long-term survival of HCC patients following hepatic resection. Numerous studies revealed that advanced age had no adverse effect on the prognosis of patients (48, 49). Meanwhile, a previous study revealed that younger age possibly was a prognostic risk factor for HCC patients as they had more advanced tumor stage and stronger tumor aggressiveness than older HCC patients (50). However, Xu et al. found that younger HCC patients tended to have a better survival outcome regardless of tumor aggressivity (51). Moreover, our meta-analysis indicated that STR was linked to worse prognosis in China and other Asian countries. However, for other Asian countries, DFS result was not statistically different, possibly due to limited sample size. Besides, studies proved that HCV is the major etiology of HCC in Japan, whereas most Chinese HCC patients have an HBV background (52, 53). The main HBV mechanisms contributing to HCC are that HBV-DNA integrates into the host genome and induces genomic instability and insertional mutagenesis of various cancer-related genes (54). However, since HCV is an RNA virus without genes integrating into the host genome, direct cellular programming and indirect inflammatory response are possible mechanisms of inducing HCC (55). Therefore, clinicopathological characteristics and prognoses of HCC caused by different viruses may differ.

We found that STR was a risk factor regardless of subgroups with low/high serum AFP concentrations. AFP, a specific tumor marker for primary HCC, is commonly employed for early screening and diagnosis of HCC; however, its specificity and sensitivity are relatively low (56). Intriguingly, numerous investigations have discovered that several serum markers may assist in diagnosing AFP negative HCC patients (57, 58). High AFP was linked to early recurrence and poor prognosis because it promoted vascular invasion and disease progression (59). Subgroup analyses of liver cirrhosis revealed that STR was a prognostic risk factor in HCC patients with or without liver cirrhosis. Recent years have witnessed a surge in research on the risk factors for HBV-cirrhosis progressing to HCC. According to relevant literature, HBV status, antiviral drugs, and liver cirrhosis severity are potential prognostic factors (60–62).

Subgroup analysis was also used to assess the effect of microvascular invasion on prognosis. The outcomes indicated that for microvascular invasion-negative patients, ruptured HCC patients exhibited a worse prognosis than non-ruptured HCC patients. However, for microvascular invasion-positive patients, whether STR correlates with a worse prognosis remains controversial. Numerous studies have confirmed that microvascular invasion is an independent risk factor for prognosis of HCC patients undergoing hepatic resection and that occult metastases caused by microvascular invasion are a major cause of HCC recurrence following surgery (63, 64). Furthermore, numerous investigations demonstrated a substantial correlation between the existence of microvascular invasion and large tumor size, high AFP concentration, and tumor localization in segment eight (65, 66). Consequently, we speculated that, in addition to the harm caused by microvascular invasion, changes in associated clinicopathological indicators (tumor size, AFP, and tumor localization) might also cause controversies in the above results. Nowadays, it is challenging to detect microvascular invasion in preoperative imaging examination, and its diagnosis still requires validation using postoperative histopathological examination (18).

The most intriguing finding of subgroup analysis was that prognosis of ruptured HCC patients after hepatic resection was opposite depending on different macrovascular invasion status (positive/negative). STR was a significant prognostic risk factor for macrovascular invasion-negative patients; nevertheless, STR was not a prognostic risk factor in macrovascular invasion-positive patients. The possible explanation for this phenomenon is that adverse STR-related prognostic influence was overshadowed by the more harmful macrovascular invasion. In Barcelona Clinic Liver Cancer (BCLC) staging systems, macrovascular invasion HCC patients are classified as an advanced stage (67). When macrovascular invasion is present, the prognosis is extremely poor, with a median survival time of 2.7 months if left untreated (68). In addition, limited included studies in macrovascular positive-subgroup analyses (OS: 4 studies; DFS: 3 studies) might be a reason. PVTT is a prevalent type of HCC macrovascular invasion. There remain numerous controversies regarding the therapeutic options for HCC patients with PVTT. According to BCLC staging system of European and American countries, HCC patients with PVTT were classified as advanced (BCLC-C) stage, and sorafenib as a palliative treatment is recommended for these patients instead of surgery or other active methods (69). However, unlike Western countries, Asia has numerous HCC patients and various treatment methods, and because each kind of HCC is unique, PVTT is not incompatible with hepatic resection (70). Numerous doctors in Asian countries continue to use active methods like surgery to treat patients with well types and liver function, and the result revealed a favorable survival benefit than non-surgical treatment in reported literature (71, 72).

Conservative treatment, TACE, and early/delayed hepatectomy are current treatments for the management of ruptured HCC (12, 13). Conservative treatment alone is suitable for ruptured HCC patients with poor baseline or extensive metastasis (8). The advantage of TACE is its high hemostasis rate, extensive indications, and it can avoid the double blow of general anesthesia and surgery (73–75). Early surgery is suitable for patients with good baseline, and due to insufficient preoperative examinations, the recurrence rate of intrahepatic tumors after surgery is high (75). Delayed hepatectomy could reduce volume of intraoperative bleeding and blood transfusion and improve better short-and long- term prognosis of ruptured HCC patients than early hepatectomy (76). Therefore, delayed surgery (after TACE achieving hemostasis) is a better treatment option for ruptured HCC patients if they are not suitable for emergent surgery.

To the best of our knowledge, this is the first meta-analysis to assess the relationship between STR and prognosis of HCC patients following hepatic resection. Besides, various subgroup analyses were performed to investigate whether the risk effect of STR varied among various subgroups. However, this study has limitations. Firstly, the included studies were retrospective, resulting in potential risks like selection and information biases. Secondly, most populations evaluated in this study were from Asia; therefore, the conclusion does not apply to Western areas with low HCC incidence. Thirdly, since the included studies were highly heterogeneous, relevant data like postoperative recurrence and complications are fully unavailable.

## Conclusions

Our study demonstrated that STR was a risk factor for long-term prognosis of HCC patients after hepatectomy. This phenomenon remained consistent in most subgroups stratified by recorded survival time, age, country, AFP concentration, liver cirrhosis, and microvascular invasion. However, STR was not associated with a worse prognosis in macrovascular invasion patients.

## Author Contributions

YC designed the research process. JX and JH searched the database for corresponding articles and drafted the meta-analysis. YW and LZ extracted useful information from the articles above. YH and YS used statistical software for analysis. BX polished this article. All authors had read and approved the manuscript and ensured that this was the case.

## Conflict of Interest

The authors declare that the research was conducted in the absence of any commercial or financial relationships that could be construed as a potential conflict of interest.

## Publisher's Note

All claims expressed in this article are solely those of the authors and do not necessarily represent those of their affiliated organizations, or those of the publisher, the editors and the reviewers. Any product that may be evaluated in this article, or claim that may be made by its manufacturer, is not guaranteed or endorsed by the publisher.

## Abbreviations

HCC: hepatocellular carcinoma
STR: spontaneous tumor rupture
TACE: transcatheter arterial chemoembolization
PVTT: portal vein tumor thrombosis
OBSs: observational studies
OS: overall survival
DFS: disease-free survival
HR: hazard ratio
CI: confidence interval
AFP: alpha-fetoprotein
NOS: Newcastle-Ottawa scale
HBV: hepatitis B virus
HCV: hepatitis C virus
HBsAg: hepatitis B surface antigen
BCLC: Barcelona clinic liver cancer.
