# Supplementary material for: Prognostic Influence of Spontaneous Tumor Rupture in Patients With Hepatocellular Carcinoma After Hepatectomy: A Meta-Analysis of Observational Studies
Source: Front Surg. 2021 Nov 16;8:769233. doi: 10.3389/fsurg.2021.769233 (PMC8635041; doi:10.3389/fsurg.2021.769233)
Supplement: Supplementary file 1 [file Table_1.DOCX]

**Supplementary Table 1. Characteristics of all the studies included in the meta-analysis.**

| Author | Year | Age(years) | | Gender(male/female) | | Tumor size(cm) | | HBV/HCV status(numbers) | | Number of tumor (solitary/multiple) | | Survival outcomes |
| --- | --- | --- | --- | --- | --- | --- | --- | --- | --- | --- | --- | --- |
|  |  | Rupture | Non-rupture | Rupture | Non-rupture | Rupture | Non-rupture | Rupture | Non-rupture | Rupture | Non-rupture |  |
| Aoki | 2014 | 820 cases≥60  340 cases<60 | 38353 cases≥60  10195 cases<60 | 873/287 | 34663/  13885 | 725 cases≥5.0  282 cases<5.0 | 10884 cases≥5.0  35237 cases<5.0 | 252/636 | 6183/33357 | 458/611 | 27348/19722 | OS |
| Chan | 2016 | 51 | 56 | NA | NA | 10.0 | 5.5 | 70/5 | 1066/47 | 52/32 | 912/342 | OS, DFS |
| Cheng | 2011 | 58 | | 707/172 | | 4.0 | | 549/285 | | NA | NA | DFS |
| Chua | 2019 | 64 | 66 | 42/7 | 86/12 | 8.5 | 7.7 | NA | NA | NA | NA | OS, DFS |
| Fan | 1999 | 53 | | 179/32 | | 8 | | NA | NA | NA | NA | OS |
| Joliat | 2018 | 61 | 68 | 14/0 | 101/25 | 7.0 | 8.0 | NA | NA | NA | NA | OS |
| Kwon | 2020 | 54 | 57 | 72/13 | 134/34 | 7.9 | 7.8 | NA | NA | 79/6 | 156/12 | OS, DFS |
| Lee | 2014 | 53 | 52 | 15/3 | 28/9 | 6.1 | 6.5 | 16/0 | 31/0 | 12/6 | 17/20 | DFS |
| Li | 2014 | 48 | 50 | 83/6 | 151/20 | 8.1 | 6.5 | 82/NA | 151/NA | NA | NA | DFS |
| Miyoshi | 2011 | 62 | 64 | 8/2 | 230/65 | 9.4 | 4.5 | NA | NA | 3/7 | 189/106 | OS |
| Mizuno | 2004 | 54 | 61 | 3/3 | 15/0 | 5.5 | 3.9 | 3/1 | 3/10 | NA | NA | OS, DFS |
| Ruan | 2020 | 51 | 53 | 48/9 | 43/14 | 8.1 | 7.1 | 29/NA | 22/NA | NA | NA | OS |
| Ruiz | 2016 | 41.9 | | 150/103 | | 14.2 | | 112/7 | | 78/175 | | OS |
| Tanaka | 2016 | 65 | 65 | 36/6 | 37/5 | 5.4 | 4.4 | 19/14 | 15/15 | 35/7 | 35/7 | OS |
| Uchiyama | 2006 | NA | NA | NA | NA | 241 cases>5.0, 790 cases≤5.0 | | NA | NA | 777/254 | | OS |
| Xiao | 2015 | 47 | | 202/32 | | 9.1 | | 217/2 | | 158/76 | | OS, DFS |
| Yang | 2013 | 49 | 51 | 127/16 | 962/128 | 8.8 | 6.4 | 128/2 | 985/27 | 97/46 | 767/323 | OS |
| Yeh | 2003 | 48 | | 164/47 | | 13.9 | | 163/16 | | NA | NA | OS |
| Zhang | 2012 | 43 | 51 | 39/2 | 353/93 | 31 cases>5.0 | 194 cases>5.0 | 35/2 | 329/7 | NA | NA | OS, DFS |
| Zhao | 2016 | 22 cases≥60, 60 cases<60 | | 73/9 | | 19 cases>10.0, 63 cases≤10.0 | | 66/NA | | 68/14 | | OS, DFS |
| Zhu | 2019 | 50 | 50 | 81/8 | 77/12 | 8.1 | 7.9 | 79/NA | 75/NA | 52/37 | 59/30 | OS, DFS |

NA, not available; HBV, hepatitis B virus; HCV, hepatitis C virus; OS, overall survival; DFS, disease-free survival.
