# Supplementary material for: Prognostic Influence of Spontaneous Tumor Rupture in Patients With Hepatocellular Carcinoma After Hepatectomy: A Meta-Analysis of Observational Studies
Source: Front Surg. 2021 Nov 16;8:769233. doi: 10.3389/fsurg.2021.769233 (PMC8635041; doi:10.3389/fsurg.2021.769233)
Supplement: Supplementary file 2 [file Table_2.DOCX]

**Supplementary Table 2.** Quality assessment of studies included.

| Author, year,  Study (Observational) | **Selection (Out of 4)** | | | | **Comparability**  **(Out of 2)** | **Outcomes (Out of 3)** | | | **Total**  **(Out of 9)** |
| --- | --- | --- | --- | --- | --- | --- | --- | --- | --- |
|  | Representativeness of exposed cohort | Selection of nonexposed cohort | Ascertainment  of exposure | Outcome not present at the start of the study |  | Assessment of outcomes | Length of follow-up | Adequacy of follow-up |  |
| Aoki, 2014 | 1 | 1 | 1 | 1 | 1 | 1 | 0 | 1 | 7 |
| Chan, 2016 | 1 | 1 | 1 | 1 | 2 | 1 | 1 | 1 | 9 |
| Cheng, 2011 | 1 | 1 | 0 | 1 | 0 | 1 | 1 | 1 | 6 |
| Chua, 2019 | 1 | 1 | 1 | 1 | 2 | 1 | 1 | 1 | 9 |
| Fan, 1999 | 1 | 1 | 0 | 1 | 0 | 1 | 1 | 1 | 6 |
| Joliat, 2018 | 1 | 1 | 1 | 1 | 2 | 1 | 1 | 1 | 9 |
| Kwon, 2020 | 1 | 1 | 1 | 1 | 2 | 1 | 1 | 1 | 9 |
| Lee, 2014 | 1 | 1 | 1 | 1 | 2 | 1 | 1 | 1 | 9 |
| Li, 2014 | 1 | 1 | 1 | 1 | 2 | 1 | 1 | 1 | 9 |
| Miyoshi, 2011 | 1 | 1 | 1 | 1 | 1 | 1 | 0 | 1 | 7 |
| Mizuno, 2004 | 1 | 1 | 1 | 1 | 2 | 1 | 0 | 1 | 8 |
| Ruan, 2020 | 1 | 1 | 1 | 1 | 2 | 1 | 0 | 1 | 8 |
| Ruiz, 2016 | 1 | 1 | 1 | 1 | 0 | 1 | 1 | 1 | 7 |
| Tanaka, 2016 | 1 | 0 | 1 | 1 | 2 | 1 | 1 | 1 | 8 |
| Uchiyama, 2006 | 1 | 1 | 1 | 1 | 0 | 1 | 1 | 1 | 7 |
| Xiao, 2015 | 1 | 1 | 1 | 1 | 0 | 1 | 1 | 1 | 7 |
| Yang, 2013 | 1 | 1 | 1 | 1 | 2 | 1 | 1 | 1 | 9 |
| Yeh, 2003 | 1 | 1 | 1 | 1 | 0 | 1 | 1 | 1 | 7 |
| Zhang, 2012 | 1 | 1 | 1 | 1 | 1 | 1 | 1 | 1 | 8 |
| Zhao, 2016 | 1 | 1 | 1 | 1 | 0 | 1 | 1 | 1 | 7 |
| Zhu, 2019 | 1 | 1 | 1 | 1 | 2 | 1 | 0 | 1 | 8 |

The observational studies were assessed by the Newcastle-Ottawa Quality Assessment Scale.
